# Perfectionism, Motives, and Barriers to Exercise from a Person-Oriented Approach

**DOI:** 10.3390/ijerph18158125

**Published:** 2021-07-31

**Authors:** María Vicent, Ricardo Sanmartín, Carolina Gonzálvez, Oswaldo Vásconez-Rubio, José Manuel García-Fernández

**Affiliations:** 1 Department of Developmental Psychology and Teaching, Faculty of Education, University of Alicante, Apdo. Correos, 99, 03080 San Vicente del Raspeig, Spain; ricardo.sanmartin@ua.es (R.S.); carolina.gonzalvez@ua.es (C.G.); josemagf@ua.es (J.M.G.-F.); 2 Faculty of Physical Culture, Central University of Ecuador, Av. Mariscal Sucre, Quito 170129, Ecuador; jhoban77@yahoo.com

**Keywords:** perfectionism, participation motives, barriers, exercise

## Abstract

Perfectionism is considered to be a significant personality factor within the sport and exercise field. However, very little is known about the reasons why individuals with different perfectionistic tendencies engage or not in physical activity. This study aims, from a person-oriented approach, to analyze if participation motives and barriers may differ among four perfectionistic profiles: *Non-Perfectionists* (low perfectionistic strivings, PS, and perfectionistic concerns, PC), *Adaptive Perfectionists* (high PS and low PC), *Maladaptive Perfectionists* (high PS and PC), and *Moderate Perfectionists* (moderate PS and PC). A sample composed of 597 (*M*_age_ = 22.08, *SD* = 3.33) undergraduates enrolled in a sport science degree from Ecuador participated in this study. *Non-Perfectionists* reported lower levels of motives, whereas *Adaptive* and *Maladaptive Perfectionists* reported higher scores on all participation motives. Significant and positive correlations were found between PS and both autonomous and controlled motives, whereas PC was positively correlated with controlled reasons and only significantly correlated with some autonomous reasons by the effect of PS. In terms of barriers, *Maladaptive Perfectionists* reported significantly higher scores on all barriers analyzed in comparison with the other three profiles, with moderate and large effect sizes. The results of the bivariate and partial correlations suggest that these inter-profile differences were explained by PC. Considering the results, it is advised to develop strategies to identify *Maladaptive Perfectionists* in order to increase their intrinsic reasons for practicing physical exercise, and to minimize their perceived barriers.

## 1. Introduction

Current societies represent a fertile ground for perfectionism. This trait of personality, considered a growing trend [1], is manifested by approximately three out of ten young people and presents important consequential outcomes in different spheres of life [2], including the exercise and sport domain. Thus, perfectionism plays a significant role in the cognition, emotions, and behaviors of exercisers and athletes, e.g., [3]. However, there is a lack of knowledge about the reasons that motivate perfectionist people to practice or to abandon sport and exercise. Thus, this study focuses on perfectionism and its relationship with motives and barriers to exercise.

Perfectionism has been defined as multidimensional, involving two higher-order dimensions commonly labeled as perfectionistic strivings (PS), which entail the need and efforts to reach perfection and to pursue extremely high performance standards; and perfectionistic concerns (PC), which capture aspects such as beliefs about perfectionistic demands, criticism from significant others, concerns over mistakes, lack of satisfaction with achievements, and self-criticism. This two-factor structure has proven to be a reliable conceptual framework to compare and understand the different studies about perfectionism, whatever the multidimensional scale of perfectionism used [4,5,6,7].

Historically, perfectionism has been considered as a transdiagnostic process involved in the development and maintenance of various forms of psychopathology [8,9]. A meta-analysis [10] supported this idea, finding that both constructs, PS and PC, are significantly related to anxiety disorders, depression, obsessive compulsive disorders, and bulimia nervosa, whereas only PS, but not PC, was significantly related to anorexia nervosa. The study also revealed that PC had the largest effect for most outcomes, except for eating disorders, in which both dimensions contributed equally.

Sport and exercise have also been areas of interest for research about perfectionism. In this context, a recent meta-analytical review proved that PC is associated with maladaptive patterns of motivation and emotion/well-being and unrelated to performance [3]. In contrast, PS is characterized by a mixed profile of adaptive and maladaptive motivation and emotion/well-being as well as better performance. The study also revealed that the negative consequences of PC were heightened when the two dimensions were examined in a partial manner (i.e., controlling the possible effects of the other dimension of perfectionism), while pure PS (i.e., PS controlling the effect of PC) emerged as a more adaptive dimension than PS. The authors concluded that PC is clearly maladaptive in the sport and exercise domain, whereas PS is a more complex and ambivalent dimension, in accordance with previous reviews [3,11,12].

However, some experts caution about this tendency of the current sport and exercise literature, mostly based on a variable-oriented approach, to present a more positive picture of perfectionism than it actually is [13]. Specifically, Flett and Hewitt [13] pointed out that, from a person-oriented approach, the potential destructiveness of perfectionism is evident for those athletes and exercisers who are characterized by extreme perfectionist profiles, warning about the existence of a “perfectionism paradox”, referring to the fact that although perfectionism is required in many sports, at the same time, it is a factor of psychological vulnerability for practitioners [14], p. 14.

Perfectionism outcomes in terms of motivation for practicing sport and exercise have been described by the scientific literature. However, research has mainly been focused on achievement motivation and self-determination theory, concluding that PS is mainly approach-oriented and self-determined (involving both ego and task goals), whereas PC is primarily controlled (sometimes even amotivated) and avoidance-oriented (see [15], for a review). Unfortunately, little is known about the factors that lead perfectionists to initiate and maintain physical activity as well as abandoning it.

### 1.1. Perfectionism and Participation Motives for Exercise

Individuals’ motives for exercise have been conceptualized as “motivational foci of physical activities” [16], p. 336, that is, participation motives describe specific reasons or goal contents for exercising [17]. People report a variety of motives for practicing physical activity such as enjoyment, social recognition, affiliation, health, managing stress, or appearance, among others [18]. Although some of these motives are predominantly intrinsically (e.g., enjoyment, challenge, and affiliation) or extrinsically regulated (e.g., weight management, appearance, and social recognition), others such as fitness and health motives are not so obviously categorized into this framework, manifesting both autonomous and controlled regulations [19,20,21,22]. 

Indeed, according to self-determination theory [23,24], different participation motives can reflect different degrees of controlled or autonomous motivation, with distinct behavioral and affective outcomes [25]. Thus, a clear distinction exists between participation motives and behavioral regulations, with the former reflecting the goal contents or the “what”, and the latter reflecting the regulatory processes or the “why” of goal pursuits [19]. In any case, the study of exercise participation motives is of great practical value because “exercise can have positive or negative effects on individuals’ psychological health, depending upon their reasons for exercising” [26], p. 107. For instance, exercising for “positive” reasons such as health and enjoyment is associated with better psychological and health-related outcomes, whereas negative reasons such as weight control and appearance are linked with poorer emotional functioning and no benefits in terms of physical well-being and exercise engagement [27].

From our knowledge, only three studies have examined the relationship between perfectionism and participation motives. Brannan et al. [26] provided data about the relationship between perfectionism and reasons for exercise in a sample of 204 female collegiate athletes from the USA. The PS dimension positively and significantly correlated with exercising to improve fitness and health, appearance, and attractiveness, and to socialize and improve mood, whereas some facets of PC (i.e., concerns over mistakes) correlated with exercising for appearance/attractiveness reasons, and to socialize and improve mood. The authors also found that concern over mistakes and appearance/attractiveness or socialization/mood management reasons for exercise increased the association between body dissatisfaction and bulimic symptoms. Taranis and Meyer [25], on their part, highlighted the link between PS and reasons for compulsive exercise (i.e., avoidance and rule-driven behavior, weight control exercise, mood improvement, lack of exercise enjoyment, and exercise rigidity) in a sample of 97 British female exercisers. Regression analyses showed that PS only positively predicted avoidance and rule-driven behavior. More recently, Madigan et al. [28] found that PC positively predicted training for avoidance of negative affect and for weight control, whereas only PS positively predicted training for mood improvement in a sample of 261 English athletes.

Taken together, and in light of self-determination theory [23,24], the results from these three studies preliminary allow guessing that PC would tend to be associated with more controlled reasons, such as avoiding negative affect, weight control, improving appearance/attractiveness, and socialization, whereas PS would be more associated with intrinsic reasons, such as improving health, fitness, and mood, but also with those controlled reasons related to body image (i.e., attractiveness and appearance).

However, these studies have several limitations that must be overcome. First of all, the results from Brannan et al. [26] and Taranis and Meyer [25], which were obtained from female samples, also need to be replicated within male populations. Secondly, the measures employed by these three studies to analyze participation motives only cover a limited number of possible reasons for practicing sport, and in the case of Taranis and Meyer [25], the motives assessed specifically referred to compulsive exercise. Hence, future studies need to evaluate other important aspects that affect the process by which a person decides to practice sport and exercise. Finally, the results derived from the three aforementioned studies are based on a variable-oriented approach. Although the variable-oriented approach has been the dominant approach in research about perfectionism, it limits the ability to make inferences about perfectionists per se (Bergman et al., 2003, cited in [29]). In contrast, “an advantage of a person-oriented approach is the inherent focus on individuals rather than variables” [30], p. 351. Additionally, because research has evidenced that both PS and PC co-exist within the individual, these studies also failed to consider the combined influence of these two dimensions [29] on reasons for exercising. In this sense, research from a person-oriented approach, which emphasizes the groups instead of dimensions, may add substantial knowledge to the relationship between perfectionism and participation motives by identifying profiles at different levels of PS and PC, which could lead to different outcomes in terms of exercise reasons, which allows “coming closer to the real person” [31], p. 348.

### 1.2. Perfectionism and Barriers to Exercise

Barriers to exercising refer to those obstacles or difficulties perceived by people in relation to physical activity, such as social physical anxiety, laziness or fatigue, lack of time, or the quality of or accessibility to facilities [32]. The study of these barriers is of great importance because it is the first step before putting into practice effective strategies to achieve a greater adherence to the initiation and maintenance of an active behavior [32].

Perfectionism, specifically PC, seems to be an important determinant of several undesirable outcomes in sport and exercise such as amotivation [33], burnout [12], and intentions to drop out [34], among others. However, although some facets of perfectionism might play a relevant role in explaining the reasons why individuals are likely to remain engaged or not in sport and exercise, the barriers or obstacles that perfectionists perceive in relation to physical activity remain unknown. Nevertheless, preliminary results suggest that those individuals with high levels of PS and PC tend to experience greater social physical anxiety [35] which may appear as an important impediment for practicing exercise. Similarly, because individuals with high levels of perfectionism strive very hard to be the best, often in various domains [36], it is possible that they find less time to practice exercise, especially when they apply their perfectionism to other areas of their life, such as studies or work. Similarly, pretending to be perfect in different domains of life can be overwhelming, which would be translated into a greater perception of fatigue.

### 1.3. This Study

**Figure 1:**
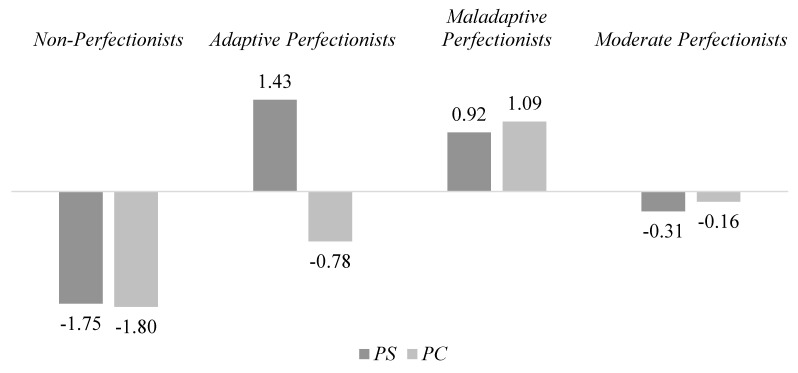
Graphic representation of the standardized average scores for the model of the latent classes (adapted from Vicent et al. [33]).

The purpose of this study is to examine the association between perfectionism and participation motives (weight and body image, fun and well-being, prevention and positive health, competition, affiliation, muscle strength and endurance, social recognition, stress control, agility and flexibility, challenge, and health emergencies) and barriers to exercising (body image/physical and social anxiety, fatigue/laziness, obligations/lack of time, and environment/facilities) from a person-oriented approach. Specifically, we will analyze whether the participation motives and barriers to exercising may differ among four perfectionism profiles previously identified by Vicent et al. [33] using latent profile analysis: *Non-Perfectionists* (low PS and PC), *Adaptive Perfectionists* (high PS and low PC)*, Maladaptive Perfectionists* (high PS and PC)*,* and *Moderate Perfectionists* (moderate PS and PC). Figure 1 shows the standardized averages on PS and PC of the four-class solution established by Vicent et al. [33]. *Moderate Perfectionists* were classified as 68% of the participants, followed by *Maladaptive Perfectionists* (21%), whereas *Adaptive* (6%) and *Non-Perfectionists* (5%) represented, respectively, 6% and 5% of the sample. This four-class solution was the most optimal model in terms of the data fit, classificatory utility, and interpretability (see [33], for a more detailed explanation of the analysis performed).

**Figure 2:**
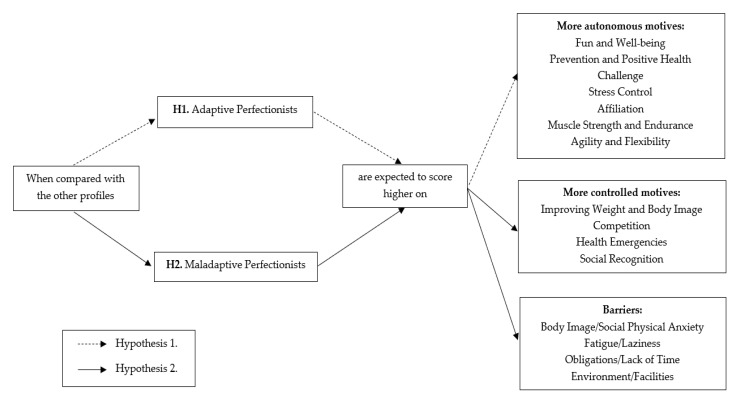
Schematic representation of the hypotheses.

In line with self-determination theory [23,24] and based on the previous literature [25,26,28], we hypothesize that (H1) *Adaptive Perfectionists* are expected to report the highest mean scores on those motives usually considered as more autonomous (i.e., fun and well-being, prevention and positive health, challenge, stress control, affiliation, and fitness-related motives) [21], whereas (H2) *Maladaptive Perfectionists* would report the highest mean scores on those participation motives usually experienced as more controlled, such as improving weight and body image, competition, health emergencies, and social recognition. Regarding the inter-profile differences in barriers to exercising, this study is largely exploratory due to the absence of preliminary research in this sense. However, considering some maladaptive outcomes of PC in the domain of sport and exercise, such as amotivation, burnout, and intentions to drop out [12,33,34], it is expected that *Maladaptive Perfectionists* perceive the largest barriers to exercising (see Figure 2 for a summary of the hypotheses).

## 2. Method

### 2.1. Participants

This sample was the same as in the study of Vicent et al. [33]. Participants were recruited by a convenience sampling technique. The initial sample consisted of 609 Ecuadorian undergraduate participants enrolled in a sport science degree program in the Central University of Ecuador. Three of the students were eliminated for having more than 60% of incomplete items, seven for presenting Z scores lower than −3 in at least one variable (univariate outliers), and two for multivariate outliers using the Mahalanobis distance [37]. Thus, the final sample was composed of 597 participants (*M*_age_ = 22.08, *SD* = 3.33). A total of 78.06% of the participants in the final sample were males (*n* = 466), whereas 21.94% of the participants were females (*n* = 131).

### 2.2. Procedure

This study was also approved by the Ethics Committee of the University of Alicante (UA-2019-12-07), and all procedures were performed following the ethical standards established in the 1964 Declaration of Helsinki and its later amendments. Written informed consent for completing questionnaires was obtained from all participants. The students were invited to respond anonymously and voluntarily to the assessment instruments in approximately 40 min. The assessment process was supervised by a duly trained research team member who explained the purpose of the survey and solved questions asked by participants.

### 2.3. Instruments

**Perfectionistic concerns and perfectionistic strivings.** A multi-measure approach, employing Hewitt’s Multidimensional Perfectionism Scale (HMPS, [38]), and Frost’s Multidimensional Perfectionism Scale (FMPS, [39]), was followed in order to assess the two higher-order perfectionism dimensions, PC and PS. Items of the FMPS are measured on a 5-point Likert scale, whereas items of the HMPS are measured on a 7-point Likert scale. A direct and back-translation method was employed to adapt both measures into the Spanish spoken in Ecuador. PC was measured using the Socially Prescribed Perfectionism (e.g., 15 items; e.g., “My family expects me to be perfect”) subscale of the HMPS, as well as the Concern over Mistakes (9 items; e.g., “I should be upset if I make a mistake”), Parental Expectations (5 items; “My parents wanted me to be the best at everything”), Parental Criticism (4 items; “My parents never tried to understand my mistakes”), and Doubts about Actions (4 items; “It takes me a long time to do something right”) subscales of the FMPS. On the other hand, PS was measured by using the Self-Oriented Perfectionism subscale (15 items; e.g., “I must work to my full potential at all times”) from the HMPS, and the Personal Standards (7 items; e.g., “I set higher goals than most people”) and Organization (6 items; e.g., “I am a neat person”) subscales from the FMPS. The selection of these subscales as indicators of PC and PS was based on results from an exploratory factor analysis (following a principal axis factoring method with varimax rotation) performed with the data at hand and previous research [4,5]. All the aforementioned subscales obtained factor loadings above 0.50, either on PC or PS. In this study, acceptable reliability coefficients were obtained for all measures employed, ranging from α = 0.70 (Parental Expectations) to α = 0.88 (Concern over Mistakes and Personal Standards).

**Participation motives.** The Self-Report on Motivation for Exercising (*Autoinforme de Motivos para la Práctica de Ejercicio Físico*; AMPEF, [40]) instrument was employed in this study. The AMPEF is a Spanish version of the Exercise Motivation Inventory-2 (EMI-2, [18]) that assesses eleven reasons for practicing sport and exercise: Weight and Body Image (7 items; e.g., “Because exercise help me to burn calories”), Fun and Well-being (5 items; “Because it makes me feel good”), Prevention and Positive Health (5 items; e.g., “To prevent health problems”), Competition (4 items; e.g., “Because I enjoy competing”), Affiliation (4 items; e.g., “To make new friends”), Muscle Strength and Endurance (4 items; e.g., “To build up my strength”), Social Recognition (4 items; e.g., “To show my worth to others”), Stress Control (3 items; e.g., “To help manage stress”), Agility and Flexibility (3 items; e.g., “To maintain flexibility”), Challenge (4 items; e.g., “To give me personal challenges to face”), and Health Emergencies (3 items; e.g., “Because my doctor advised me to exercise”). Items are measured on a 10-point Likert scale. The scale was reviewed by two Ecuadorian experts who verified the adequacy of the items’ wording in Ecuadorian Spanish. In this study, the reliability coefficients of the eleven subscales were higher than 0.76 in all cases, with the exception of Health Emergencies (α = 0.67).

**Barriers to exercise.** The Self-Report on Barriers to Exercising (*Autoinforme de Barreras para la Práctica de Ejercicio Físico*; ABPEF, [32]) instrument was used in this study to analyze four types of reasons that prevent practicing exercise: Body Image/Social Physical Anxiety (5 items; e.g., “Feeling that my physical appearance is worse than that of others”), Fatigue/Laziness (6 items; e.g., “Lack of will to be consistent”), Obligations/Lack of Time (3 items; e.g., “Having too much work”), and Environment/Facilities (3 items; e.g., “Being too far from where I can exercise”). Items are measured on a 10-point Likert scale. The scale was also reviewed by two Ecuadorian experts who verified the adequacy of the items’ wording in Ecuadorian Spanish. In this study, the reliability coefficients of the subscales ranged from α = 0.72, for Fatigue/Laziness, to α = 0.90, for Body Image/Social Physical Anxiety.

### 2.4. Data Analysis

First, descriptive analysis including means, standard deviations, and bivariate and partial correlations (controlling the effect of the other perfectionism dimension) between perfectionism dimensions, i.e., PC and PS, and all subscales of the AMPEF and ABPEF was conducted. Bivariate (shared) correlations must be interpreted in terms of why an individual with high levels in one dimension differs or not from another individual with low levels in the same dimension. In contrast, partial (unique) correlations allow acknowledging the “pure” relationship between one perfectionism dimension and the other variables studied, by keeping the other perfectionism dimension constant and controlling its effect on the dimension being analyzed [41,42]. The effect size of these correlations was interpreted in accordance with Cohen’s criteria: small (*r* = 0.10 and 0.30), moderate (*r* = 0.30 and 0.50), and large (*r* ≥ 0.50) [43].

Secondly, based on the four profiles previously identified by Vicent et al. [33] by latent profile analysis (see Figure 1), the inter-profile differences in the scores on the subscales of the AMPEF and ABPEF were analyzed using analysis of variance (ANOVA). Post hoc tests, following the Bonferroni method, were performed to identify between which profiles there were statistically significant differences. Additionally, the effect size of the observed differences was calculated using Cohen’s *d* index. Specifically, *d* levels between 0.20 and 0.49 indicate a small effect magnitude; levels between 0.50 and 0.79 indicate a moderate magnitude; and levels ≥0.80 indicate a large magnitude [43].

## 3. Results

### 3.1. Descriptive Statistics

Means, standard deviations, reliability coefficients, and bivariate and partial correlations for all the variables in the study are presented in Table 1.

Positive and statistically significant bivariate correlations were found between PS and all participation motives and barriers examined, apart from Fatigue and Laziness, whose correlation coefficient did not reach statistical significance. The magnitudes of these correlations were of a small effect size in all cases except for Social Recognition, which was moderate. Similarly, PC also positively and significantly correlated with all barriers and participation motives examined, with the exception of the non-significant correlations between PC and the Fun and Well-Being, and Prevention and Positive Health motives. Effect sizes were of a small magnitude for most cases. However, moderate correlations were obtained for the relationship between PC and Social Recognition, as well as for two of the barriers analyzed (Fatigue/Laziness and Environment/Facilities). Correlations of a large size were observed between PC and Body Image/Physical and Social Anxiety.

Results of partial correlations between each perfectionistic dimension (controlling the effect of the other dimension) and the participation motives and barriers to exercising showed positive and significant correlations between PS and all participation motives, except for Health Emergences, as well as negative and significant correlations with all barriers, except for Obligation/Lack of Time. Effect sizes associated with these correlations were of a small magnitude. Regarding the PC dimension, positive and significant correlations were obtained for Weight and Body Image, Competition, Social Recognition, and Health Emergencies, as well as for the four barriers to exercising studied. In contrast, PC negatively and significantly correlated with Fun and Well-being, and Prevention and Positive Health. Again, small effect sizes were found for many of these associations, although in the case of barriers, correlations of a moderate magnitude were found for Fatigue/Laziness and Environment/Facilities, as well as correlations of a large magnitude for Body Image/Physical and Social Anxiety.

### 3.2. Inter-Profile Differences

The results of the ANOVA to examine the mean differences between the four perfectionism profiles on participation motives and barriers to exercise are presented in Table 2. Significant inter-profile differences were found for all motives and barriers analyzed. *Non-Perfectionists* reported the lowest mean scores on all variables studied excluding Fatigue/Laziness, and Environment/Facilities, whose lowest levels were reported by the *Adaptive Perfectionists*. In contrast, *Adaptive Perfectionists* reported the highest scores on Fun and Well-being, Prevention and Positive Health, Muscle Strength and Endurance, and Challenge, whereas *Maladaptive Perfectionists* reported the highest mean scores on the rest of the participation motives and on all barriers.

As it can be seen in Table 3, when examining post hoc comparisons, *Non-Perfectionists* reported significantly lower levels of participation motives, except for Affiliation and Health Emergencies, in comparison with *Adaptive Perfectionists*, with moderate and large effect sizes (*d* = 0.54–0.86). Similarly, the *Non-Perfectionists* profile had significantly lower scores on all motives for exercise than *Maladaptive* and *Moderate Perfectionists*, excluding Fun and Well-being, and Prevention and Positive Health (in both), and Affiliation, Stress Control, and Challenge (in *Moderate Perfectionists*). The magnitude of these differences was moderate and large, ranging from *d* = 0.52 to 1.67. The contrasts between *Adaptive Perfectionists* and *Maladaptive* and *Moderate Perfectionists* only reach statistical significance for Social Recognition, in the case of *Maladaptive Perfectionists*, and for Prevention and Positive Health, in the case of *Moderate Perfectionists*. In both comparisons, *Adaptive Perfectionists* reported the lowest scores, with moderate effect sizes (*d* = 0.63 and 0.67, respectively). In contrast, comparisons between *Maladaptive Perfectionists* and *Moderate Perfectionists* were significant, but of a small and moderate magnitude (*d* = 0.27–0.61), in all motives examined, excluding Fun and Well-Being, and Affiliation. In this case, *Maladaptive Perfectionists* scored higher than *Moderate Perfectionists* in the motives examined.

Regarding post hoc contrasts on barriers to exercise, *Maladaptive Perfectionists* reported significantly higher mean scores on all barriers analyzed in comparison with the other three profiles. The larger magnitudes were found when *Maladaptive Perfectionists* were compared with the *Non-Perfectionists* (*d* = 0.93–1.51) and *Adaptive Perfectionists* (*d* = 0.78–1.14) profiles. *Moderate Perfectionists* also scored significantly higher than *Non-Perfectionists* (in all the barriers) and *Adaptive Perfectionists* (with the exception of Obligations and Lack of Time). Finally, non-significant differences were found between *Non-Perfectionists* and *Adaptive Perfectionists* in terms of barriers to exercise.

## 4. Discussion

The purpose of this study was to determine, from a person-oriented approach, whether different profiles of perfectionism (i.e., *Non-Perfectionists*, *Maladaptive Perfectionists*, *Adaptive Perfectionists*, and *Moderate Perfectionists*) differed with respect to the reasons for and barriers to practicing physical activity. Additionally, following Stoeber’s [44] suggestion, the shared (bivariate correlations) and unique (controlled correlations) relationships between PC, PS, and participation motives and barriers were analyzed in order to explain which of these dimensions was responsible for the inter-profile differences found.

### 4.1. Perfectionism and Participation Motives

In respect of the participation motives, *Maladaptive Perfectionists* reported the highest levels on Weight and Body Image, Competition, Affiliation, Social Recognition, Stress Control, Agility and Flexibility, and Health Emergencies, whereas *Adaptive Perfectionists* reported the highest scores on Fun and Well-Being, Prevention and Positive Health, Muscle Strength and Endurance, and Challenge. These results are in line with our expectations and previous research [25,26,28], as most of the reasons for which *Maladaptive Perfectionists* scored higher are considered to be more controlled (i.e., Weight and Body Image, Competition, Social Recognition, Agility and Flexibility, and Health Emergencies), whereas *Adaptive Perfectionists* seemed to be more linked with autonomous reasons (Fun and Well-Being, Prevention and Positive Health, and Challenge). However, it is worth noting that these differences did not reach statistical significance for all comparisons. Therefore, our hypotheses are partially supported, because, according to the results, *Adaptive* and *Maladaptive Perfectionists* manifested similar levels of participation motives for exercising, regardless of whether they are more or less self-determined reasons, with the only exception of Social Recognition. In respect of Social Recognition, it is important to remember that *Maladaptive Perfectionists* are defined by high levels of both PS and PC, whereas *Adaptive Perfectionists*, in contrast, are defined by high levels of PS but low levels of PC [33]. Thus, the key difference between these two profiles is the degree of PC, which entails, between others, interpersonal facets of perfectionism, such as Socially Prescribed Perfectionism, Parental Expectations and Parental Criticism [4,5,6]. These three perfectionism dimensions are characterized by the belief that the environment is highly demanding of perfectionism as well as critical [31,39]. Thus, considering this inherent desire of *Maladaptive Perfectionists* to meet others’ requirements as well as avoiding their disapproval, it is therefore not surprising that seeking praise from peers and friends is an important reason for practicing sport and exercise, when compared with the other profiles with milder forms of PC. Unfortunately, this need of approval is not considered as a positive reason for exercising. According to the results of Maltby and Day [45], who analyzed the impact of thirteen participation motives and different measures of psychological adjustment (self-esteem, somatic symptoms, anxiety, social dysfunction, and depression), social recognition showed one of the most negative patterns of correlations in the short and long term.

Despite the scarce differences between *Adaptive* and *Maladaptive Perfectionists* in participation motives, both profiles significantly reported higher levels on most of the reasons analyzed in comparison with *Non-Perfectionists*. Overall, this means that there are many reasons why perfectionists, either *Adaptive* or *Maladaptive*, consider it important to exercise compared to *Non-Perfectionists*. These differences were of a moderate and large effect size, which means that they not only are interesting from a theoretical point of view but also have a considerable impact on real life [46]. Although this fact might be understood as a positive outcome for *Maladaptive Perfectionists*, these findings should be interpreted with caution because not all reasons for exercising are equally valuable [27]. In this sense, *Maladaptive Perfectionists* did not differ from *Non-Perfectionists* regarding some of those motives considered more autonomous or self-determined (i.e., Fun and Well-Being, and Prevention and Positive Health), whereas the higher differences between both profiles were reported for those motives considered more external or controlled, such as Weight and Body Image, Competition, or Social Recognition. It should be noted that reasons for exercise focused on weight control and physical appearance have been associated with body dissatisfaction and disordered eating behaviors [26,47]. Moreover, although external motives can be useful under certain circumstances, individuals engaged in exercise by choice and for intrinsic reasons will experience more task perseverance and psychological well-being, as well as less stress, anxiety, and self-criticism [48]. Consequently, *Adaptive Perfectionists* would experience more strong reasons for exercising by Fun and Well-Being (an archetypical intrinsic reason), and Prevention and Positive Health than *Non-Perfectionists*. Therefore, when comparing these two profiles of perfectionism, *Adaptive* and *Maladaptive*, with the *Non-Perfectionists*, the *Adaptive* profile would present greater advantages.

This idea was also supported by the correlational analysis, as only PS reported shared and unique positive correlations with all reasons, either controlled or autonomous (except for Health Emergencies), manifesting an ambiguous pattern [3,11,12,49]. In contrast, PC was more clearly maladaptive [3,11,12,49], as it showed unique positive correlations with reasons considered more controlled (Weight/Body Image, Competition, Social Recognition, and Health Emergencies), and negative correlations with some autonomous reasons, such as Fun and Well-being, and Prevention and Positive Health, whereas its positive relationships with all fitness reasons and other autonomous reasons such as Challenge, Stress Control, and Affiliation were only significant by the effect of PS.

Moreover, the results can also be interpreted in light of the dual process theory of perfectionism which establishes that PC is mainly avoidance-oriented, whereas PS is mainly approach-oriented [50]. An example of this dual process would be the relationship between the two perfectionism dimensions and the health-related scales: Prevention/Positive Health (approach-oriented) and Health Urgencies (avoidance-oriented). According to our findings, PC was more strongly associated with Health Pressures which reflects the need to exercise as a treatment or alleviation of a diagnosed health disorder, whereas PS was more strongly related with Prevention and Positive Health which has more positive connotations reflecting the desire of exercising as part of an active and healthy lifestyle [40]. In this regard, future studies might explore if the approach and avoidance orientations may mediate the relationship between perfectionism and participation motives.

### 4.2. Perfectionism and Barriers to Exercise

In terms of barriers to exercise, PC showed positive and significant shared and unique correlations with the four barriers analyzed. In contrast, PS showed positive shared correlations with all barriers, except for Fatigue and Laziness (which was non-significant), and negative unique relationships with all barriers, except for Obligations and Lack of Time (which was also non-significant).

These correlational results are consistent with those obtained from a person-oriented approach, as *Non-Perfectionists* (low PS and PC) and *Adaptive Perfectionists* (high PS and low PC) appeared not to differ in terms of barriers to exercising, showing the lowest levels. In contrast, *Maladaptive Perfectionists* (high PS and PC) scored significantly higher on all barriers in comparison with the other three profiles, reporting large effect sizes for most of the comparisons with the *Non-Perfectionists* and *Adaptive Perfectionists* profiles. Unfortunately, our results cannot be compared with any previous research as this is the first study addressing the relationship between perfectionism and barriers to exercising. However, these findings are in line with our expectations since PC involves difficulties in exercise engagement, as it is positively associated with amotivation, burnout, and intentions to drop out [12,33,34]. Hence, it is not surprising that those perfectionists characterized by high levels of PC, i.e., *Maladaptive Perfectionists*, perceive more obstacles for practicing exercise, such as social physical anxiety, fatigue and laziness, lack of time or difficulties in organizing their free time, and deficiencies in or poor accessibility to spaces where exercise is practiced.

A special mention features the barrier relative to Body Image/Physical and Social Anxiety because PC was more closely correlated with this than with any other barrier, and therefore Body Image/Physical and Social Anxiety was the barrier for which the greatest differences between the *Maladaptive* profile and the rest of the profiles were observed. High levels on Body Image/Physical and Social Anxiety indicate that the embarrassment of showing one’s body in public, or the fear over evaluations, jokes, or criticism about one’s body, is an important impediment to exercise. These beliefs are related to social physique anxiety [51], which has been associated, in turn, with negative behavioral and health-related outcomes in the context of physical activity [52]. Moreover, in line with our results, previous research has found that the combination of high levels on both PS and PC reported higher levels on social physique anxiety than any other possible combination [35]. However, results from our correlational analyses suggest that this strong association between *Maladaptive Perfectionists* and Body Image/Physical and Social Anxiety barriers would be mainly explained by PC, as well as by the effect that this dimension has on PS. Individuals with high PS tend to pursue unrealistically high standards [4,5,6] that might also be applied to the domain of physical appearance. On the other hand, PC is characterized by concerns over perfectionistic criticisms from others as well as a huge fear over mistakes and not reaching others’ expectations [4,5,6]. Hence, when high levels on both dimensions are combined, perfectionists might avoid practicing exercise to prevent others’ judgements about their supposed physique imperfections or flaws in relation to thinness, shape, or musculature; this might be especially relevant in the case of those physical activities in which the body is more exposed, such as swimming. In this sense, future research should analyze if perfectionists might perceive different barriers depending on the type of exercise and sport performed.

### 4.3. Limitations and Future Research

Several limitations of the current study should be taken into account. First, the sample of this study was composed of undergraduate students enrolled in a sport science degree in Ecuador, and the actual engagement level of each study participant in physical activity was not collected. Therefore, the results should be generalized with caution to professional athletes. Furthermore, considering that the type of barriers to exercise might vary considerably depending on different aspects, such as age (e.g., [53]) or socioeconomic or cultural factors, especially on the resources and facilities available (e.g., [54,55,56]), future research should test if the findings of our study can be applied to other age samples and cultures. Moreover, it must be noted that females were underrepresented in our study as only 21.94% of the participants were female. Additionally, gender differences in terms of motives and barriers to exercise have been well established (e.g., [57,58]). Therefore, it should be interesting to examine if similar inter-profile differences in terms of motives and barriers would be obtained or not when males and females are analyzed separately. Additionally, the results of this study cannot be interpreted in terms of causality due to its cross-sectional design. Future research needs to address if there exist causality relationships between perfectionism and participation motives and barriers to exercise by using longitudinal and experimental methods. Lastly, although the labels adaptive and maladaptive have been employed in this study and previous research on perfectionism from a person-oriented approach (e.g., [33,59,60,61,62], it is important to take into account that the issue about whether these profiles are functional or dysfunctional must be an empirical question. Thus, these labels should be employed with caution when other outcomes, different from reasons for and barriers to exercise, are being examined among perfectionism profiles.

## 5. Conclusions and Practical Implications

Despite the limitations, there are two notable features of this research. On the one hand, this is the first study that has analyzed the relationship between perfectionism and barriers to exercise. Furthermore, this study contributes to enriching the limited existing literature [25,26,28] about the link between perfectionism and participation motives, analyzing a greater diversity of reasons for exercise.

On the other hand, the person-oriented approach together with the analysis of bivariate and partial correlations adopted in this study permits establishing inferences not only about how the perfectionism dimensions (PS and PC) are related to participation motives and barriers to exercise but also about the reasons and obstacles perceived by individuals with different perfectionism profiles, resulted from the combination of different levels of PS and PC. It thereby allows a better understanding of perfectionism at both the individual and the dimension level.

Overall, the results evidence that PC is more closely correlated with more controlled motives (i.e., Weight and Body Image, Competition, Social Recognition, and Health Emergencies) and perceived barriers to exercise, although it also displays some autonomous motives (i.e., Challenge, Affiliation, Stress Control, and fitness-related reasons) when the effect of PS is not controlled. In contrast, PS seems to be more ambiguous, as it shows both shared and unique positive relationships with controlled and autonomous motives, as well as negative unique relationships with barriers which became positive by the effect of PC. From a person-oriented focus, except in the case of Social Recognition, *Adaptive* (high PS and low PC) and *Maladaptive Perfectionists* (high PS and PC) do not manifest significant differences between them in terms of motives. However, both profiles reported significantly higher participation motives than *Non-Perfectionists,* with some exceptions that did not reach statistical significance. The results evidence that both *Adaptive* and *Maladaptive Perfectionists* engage in physical activity for a wide range of reasons, combining those considered more autonomous and desirable with others that are more controlled and undesirable. However, when barriers are considered, important differences appear between these two profiles, as individuals classified as *Maladaptive Perfectionists* perceived significantly more difficulties and impediments for practicing physical activity in comparison with any other profile, whereas *Adaptive* and *Non-Perfectionists* reported the lowest levels of perceived barriers.

It is a well-known fact that regular physical exercise ensures benefits at a social, mental, and physical level [57]. Consequently, increasing physical activity has become an important social issue by developing effective policies and strategies aimed at not only stimulating it but also removing the barriers that prevent it, as well as identifying which factors lead individuals to initiate, maintain, and/or abandon exercise [53]. According to our results, perfectionism is an important factor that might influence the reasons why individuals decide to exercise or not. Therefore, professional trainers, coaches, and physical educators should strive to identify those practitioners considered *Maladaptive Perfectionists*, a profile that characterized 2 out of 10 exercisers, approximately [33]. This is because these practitioners would be more motivated for inappropriate (more controlled) reasons as well as perceiving more barriers to exercise. In this sense, because, as experts assure, intrinsic motivation is “quality” motivation [48], intrinsic reasons for exercise such as fun and well-being must be enhanced and promoted, especially in those perfectionists who manifest high levels of PC (i.e., *Maladaptive Perfectionists*). Furthermore, it would be advisable to implement strategies focused on minimizing the barriers perceived by *Maladaptive* and *Moderate Perfectionists* as well as reducing their levels of PC given that it would implicate a greater perception of obstacles to and difficulties in practicing exercise, being, in turn, a risk factor for inadequate exercise adherence. Special attention should be directed to perceived body image/physical and social anxiety obstacles, particularly important barriers for *Maladaptive Perfectionists*. Thus, *Maladaptive Perfectionists*’ exercise might benefit from specific strategies addressed to monitoring, preventing, and reducing their social physique anxiety.

## Figures and Tables

**Table 1:** Reliability, means, standard deviations, and bivariate and partial correlations between the perfectionism dimensions and the participation motives and barriers to exercising.

		Bivariate Correlations		Partial Correlations		α	*M*	*SD*
		PS	PC	PS	PC			
AMPE	Weight/Body Image	0.26 **	0.24 **	0.18 **	0.14 **	0.86	47.29	14.99
	Fun/Well-being	0.18 **	−0.02	0.21 **	−0.11 *	0.82	41.08	8.17
	Prevention/Positive Health	0.19 **	−0.03	0.23 **	−0.13 *	0.85	42.08	7.64
	Competition	0.26 **	0.23 **	0.19 **	0.13 *	0.83	25.51	10.21
	Affiliation	0.14 **	0.09 *	0.11 *	0.04	0.79	26.91	8.92
	Muscle Strength/Endurance	0.26 **	0.11 **	0.23 **	−0.01	0.83	31.84	7.26
	Social Recognition	0.32 **	0.36 **	0.19 **	0.26 **	0.78	22.98	9.96
	Stress Control	0.20 **	0.09 *	0.18 **	0.00	0.77	23.01	5.86
	Agility/Flexibility	0.22 **	0.16 **	0.17 **	0.07	0.79	22.13	6.25
	Challenge	0.24 **	0.11 **	0.21 **	0.00	0.80	31.28	7.56
	Health Emergencies	0.13 **	0.24 **	0.02	0.20 **	0.67	15.58	7.91
ABPE	Body Image/Physical and Social Anxiety	0.13 **	0.55 **	−0.15 **	0.56 **	0.90	15.90	13.05
	Fatigue/Laziness	0.08	0.44 **	−0.14 **	0.45 **	0.85	24.25	14.01
	Obligations/Lack of time	0.10 *	0.30 **	−0.04	0.29 **	0.72	13.65	7.42
	Environment/Facilities	0.10 *	0.46 **	−0.13 *	0.47 **	0.80	11.71	8.01
*M*		90.70	97.27	---	---	---	---	---
*SD*		15.01	17.48	---	---	---	---	---

*Note*: AMPE = The Self-Report on Motivation for Exercising, ABPE = The Self-Report on Barriers to Exercising, PS = perfectionistic strivins, PC = perfectionistic concerns. * *p* < 0.05, ** *p* < 0.01.

**Table 2:** Multivariate analysis of variance (ANOVA) of participation motives and barriers to exercising by perfectionism profiles.

AMPE	*Non-Perfectionists*		*Adaptive Perfectionists*		*Maladaptive Perfectionists*		*Moderate Perfectionists*		Statistical Significance and Effect Sizes		
	*M*	*SD*	*M*	*SD*	*M*	*SD*	*M*	*DT*	*F_(3, 593)_*	*p*	η^2^
Weight/Body Image	34.45	20.72	49.51	15.56	52.72	13.79	46.45	14.01	15.37	<0.001	0.07
Fun/Well-being	38.39	9.96	44.48	6.65	41.79	8.30	40.83	8.00	3.34	0.019	0.02
Prevention/PH	41.18	7.982	46.55	5.12	43.46	6.77	41.39	7.87	6.11	<0.001	0.03
Competition	17.27	11.36	26.79	10.89	28.83	9.30	25.03	9.92	12.87	<0.001	0.06
Affiliation	23.09	10.33	25.79	10.71	28.54	8.59	26.79	8.67	3.69	0.012	0.02
Muscle Strength/E	27.09	10.15	34.44	6.10	33.96	6.34	31.37	7.08	10.67	<0.001	0.05
Social Recognition	13.48	8.99	22.00	12.51	27.89	8.55	22.26	9.49	23.66	<0.001	0.11
Stress Control	20.24	8.17	24.24	6.44	24.45	5.75	22.69	5.52	6.04	<0.001	0.03
Agility/Flexibility	18.48	7.42	22.68	6.05	24.11	5.63	21.76	6.17	8.95	<0.001	0.04
Challenge	28.15	8.75	33.34	8.66	33.18	7.20	30.79	7.35	6.04	<0.001	0.03
Health Emergencies	11.45	8.87	13.65	9.28	17.65	8.13	15.40	7.49	6.75	<0.001	0.03
ABPE											
Body Image/PSA	4.00	6.61	4.65	7.42	23.60	14.09	15.22	11.85	37.48	<0.001	0.16
Fatigue/Laziness	15.21	10.69	15.00	11.97	31.17	15.18	23.44	12.96	21.80	<0.001	0.10
Obligations/LT	9.48	7.82	10.79	6.67	16.48	7.39	13.29	7.15	12.09	<0.001	0.06
Environment/F	7.33	6.89	6.58	6.01	15.59	8.52	11.19	7.53	19.57	<0.001	0.09

*Note*: AMPE = The Self-Report on Motivation for Exercising, ABPE = The Self-Report on Barriers to Exercising, PS = perfectionistic standards, PC = perfectionistic concerns, PH = Positive Health, E = Endurance, PSA = Physical and Social Anxiety, LT = Lack of Time, F = Facilities.

**Table 3:** Cohen’s d indexes for post hoc contrasts between the mean scores obtained by the four profiles on participation motives and barriers to exercise.

AMPE	*Non-P.* *vs.* *Adaptive P.*	*Non-P.* *vs.* *Maladaptive P.*	*Non-P.* *vs.* *Moderate P.*	*Adaptive P.* *vs.* *Maladaptive P.*	*Adaptive P.* *vs.* *Moderate P.*	*Maladaptive P.* *vs.* *Moderate P.*
Weight/Body Image	0.81 **	1.18 ***	0.82 ***	-	-	0.45 ***
Fun/Well-being	0.71 *	-	-	-	-	-
Prevention/PH	0.79 *	-	-	-	0.67 **	0.27 *
Competition	0.85 **	1.19 ***	0.77 ***	-	-	0.39 **
Affiliation	-	0.61 *	-	-	-	-
Muscle Strength/E	0.86 **	0.95 ***	0.58 **	-	-	0.28 **
Social Recognition	0.79 **	1.67 ***	0.93 ***	0.63*	-	0.61 ***
Stress Control	0.54 *	0.67 **	-	-	-	0.32 *
Agility/Flexibility	0.62 *	0.93 ***	0.52 **	-	-	0.39 **
Challenge	0.57 *	0.66 **	-	-	-	0.33 *
Health Emergencies	-	0.75 ***	0.52 **	-	-	0.29 *
ABPE						
Body Image/PSA	-	1.51 ***	0.97 ***	1.14 ***	0.91 ***	0.67 ***
Fatigue/Laziness	-	1.11 ***	0.64 **	1.10 ***	0.65 **	0.57 ***
Obligations/LT	-	0.93 ***	0.53 *	0.78 **	-	0.44 ***
Environment/F	-	1.01 ***	0.52 *	1.10 ***	0.62 *	0.57 ***

*Note*: AMPE = The Self-Report on Motivation for Exercising, ABPE = The Self-Report on Barriers to Exercising, PS = perfectionistic standards, PC = perfectionistic concerns, PH = Positive Health, E = Endurance, PSA = Physical and Social Anxiety, LT = Lack of Time, F = Fatigue. * *p* < 0.05. ** *p* < 0.01. *** *p* < 0.001.

## Data Availability

The data presented in this study are available on request from the corresponding author.
